# Value-driven modulation of visual perception by visual and auditory reward cues: The role of performance-contingent delivery of reward

**DOI:** 10.3389/fnhum.2022.1062168

**Published:** 2022-12-23

**Authors:** Jessica Emily Antono, Roman Vakhrushev, Arezoo Pooresmaeili

**Affiliations:** Perception and Cognition Lab, European Neuroscience Institute Göttingen–A Joint Initiative of the University Medical Center Göttingen and the Max-Planck-Society, Göttingen, Germany

**Keywords:** visual perception, reward, pupil response, sensory modality, psychophysics

## Abstract

Perception is modulated by reward value, an effect elicited not only by stimuli that are predictive of performance-contingent delivery of reward (PC) but also by stimuli that were previously rewarded (PR). PC and PR cues may engage different mechanisms relying on goal-driven versus stimulus-driven prioritization of high value stimuli, respectively. However, these two modes of reward modulation have not been systematically compared against each other. This study employed a behavioral paradigm where participants’ visual orientation discrimination was tested in the presence of task-irrelevant visual or auditory reward cues. In the first phase (PC), correct performance led to a high or low monetary reward dependent on the identity of visual or auditory cues. In the subsequent phase (PR), visual or auditory cues were not followed by reward delivery anymore. We hypothesized that PC cues have a stronger modulatory effect on visual discrimination and pupil responses compared to PR cues. We found an overall larger task-evoked pupil dilation in PC compared to PR phase. Whereas PC and PR cues both increased the accuracy of visual discrimination, value-driven acceleration of reaction times (RTs) and pupillary responses only occurred for PC cues. The modulation of pupil size by high reward PC cues was strongly correlated with the modulation of a combined measure of speed and accuracy. These results indicate that although value-driven modulation of perception can occur even when reward delivery is halted, stronger goal-driven control elicited by PC reward cues additionally results in a more efficient balance between accuracy and speed of perceptual choices.

## Introduction

Stimuli associated with rewards have a strong influence on our behavior as they trigger the expectation of desirable outcomes, thereby driving agents to optimize their goal-directed actions (Schultz, 2015) and value-based choices (Delgado, 2007; Wallis, 2007; Schultz, 2015). Accordingly, brain areas underlying action planning and value-based decisions are strongly modulated by rewards. Moreover, reward effects even extend to the earliest stages of information processing in the brain as reward associations of stimuli influence their representation in the primary sensory areas (Shuler and Bear, 2006; Serences, 2008). Understanding the underlying mechanisms of value-driven modulation of perception is important since it allows a better understanding of how experience-related and contextual factors in general influence sensory perception (Pessoa and Engelmann, 2010; Seriès and Seitz, 2013).

Reward effects on perception are typically investigated using paradigms where correct detection or discrimination in a perceptual task (Engelmann and Pessoa, 2007) or efficient orienting responses in a motor task (Milstein and Dorris, 2007) lead to higher magnitude or probability of rewards. In such scenarios, prioritization of reward cues, through engaging mechanisms such as selective attention or preparation of oculomotor responses, aligns with the goal-driven mechanisms that help agents to maximize their obtained rewards (Chelazzi et al., 2013; Failing and Theeuwes, 2018). Using such tasks, value-driven modulations have been observed at the early stages of sensory processing in the brain. For instance, Weil et al. (2010) provided evidence that rewarding feedbacks improved behavioral performance in a visual discrimination task and also increased the activity in the human primary visual cortex during the discrimination phase following a reward feedback. Another study by Pleger et al. (2008) also demonstrated that reward facilitated somatosensory judgments. There, high reward cues improved tactile performance and enhanced the hemodynamic response in the primary somatosensory cortex, indicating that reward signals can influence early sensory areas when a decision is based on the sensory features of stimuli. Thus, reward signals, during the delivery of reward or during the presentation of reward-predicting cues, can be propagated not only within the classical reward-related regions, but also to sensory areas, especially when the reward delivery is contingent on the accuracy of sensory judgments [i.e., performance-contingent (PC)]. One criticism to these designs is that value-driven effects cannot be distinguished from attentional (Maunsell, 2004) or cognitive control mechanisms (Botvinick and Braver, 2015) that are involved in processing of the task-relevant feature of a task. Accordingly, such paradigms do not allow a differentiation between value-driven effects due to voluntary, goal-driven mechanisms from effects due to stimulus-driven and involuntary mechanisms.

Another line of research has shown that value-driven modulation of perception also occurs when reward cues are not the relevant feature of the task or when reward delivery and hence the motivation to strategically optimize performance has been removed. For instance, the delivery of reward in response to a saccadic target in some trials can affect the oculomotor performance in subsequent unrewarded trials when a non-target stimulus contains a similar feature as the rewarded target in the past (Hickey and van Zoest, 2012). It has also been shown that reward effects outlast the delivery of reward so that previously rewarded (PR) features automatically affect participants’ performance (Yantis et al., 2012; De Tommaso et al., 2017). The latter experiments typically employ a two-phase paradigm (De Tommaso and Turatto, 2021), where in the first training or conditioning phase participants learn the association of stimulus features with certain amount or probability of reward, and in the subsequent test phase PR cues are presented without the actual delivery of reward (i.e., during extinction). Although during the test phase reward associated cues are not reinforced anymore, it has consistently been shown that they can still involuntarily capture participants’ attention, a phenomenon called value-driven attentional capture (VDAC) (Anderson et al., 2011), and thereby influence perceptual judgments across a variety of tasks (Anderson et al., 2011; Yantis et al., 2012; Camara et al., 2013; Failing and Theeuwes, 2015; Bucker and Theeuwes, 2017; Tankelevitch et al., 2020). The typical finding of these studies is that when PR stimuli are the same as the target of a task they facilitate performance (accuracy or RT) but importantly when they are irrelevant to the task or assigned to distractors, they can impair performance (Anderson et al., 2014; Asutay and Västfjäll, 2016; Gong et al., 2017; Bucker and Theeuwes, 2018; Qin et al., 2020; Watson et al., 2020), a so-called value-driven distraction (Rusz et al., 2020). Such effects likely arise as a result of the enhanced representation of distractors in visual cortex (Itthipuripat et al., 2019), which limit the processing resources that are available to the target.

Interestingly, it is not always the case that task-irrelevant reward cues capture attention away from the target and suppress performance. For instance, Pooresmaeili et al. (2014) utilized one sensory modality (audition) to signal the reward value while keeping the target of the task in another modality (vision). Using this design, it was shown that task-irrelevant auditory cues that were previously associated with high reward enhanced the visual sensitivity compared to low reward cues. A follow-up study (Vakhrushev et al., 2021) used a similar design and compared task-irrelevant reward cues from the same (vision) or different (audition) sensory modality in terms of their effect on perceptual decisions made about a visual target. In this study, it was found that PR auditory and visual cues had distinct effects on behavioral and electrophysiological correlates of visual perception, suggesting that reward-driven modulations may have dependencies on the sensory modality of task-irrelevant stimuli.

Overall, across different paradigms employed to investigate the effects of reward on sensory perception, PC rewards have been often found to be associated with the facilitation of sensory processing, whereas divergent effects were observed for cues previously associated with rewards based on whether the target or the task-irrelevant distractors contained a rewarded feature. Another factor that also seems to weigh in is where the reward information was signaled from, with different effects for rewards cued intra-modally or cross-modally. However, a systematic investigation of these factors where the same perceptual judgment is tested under different modes of reward delivery and cuing has been missing. Therefore, in the current study, we designed a paradigm that tested the effect of three factors on visual perception: reward magnitude, sensory modalities of reward cues, and the contingency of reward delivery on task performance. Specifically, a similar design as two previous studies from our lab (Pooresmaeili et al., 2014; Vakhrushev et al., 2021) was used where auditory or visual cues were first associated with either high or low monetary reward during a training phase (referred to as conditioning). During the test phase, auditory and visual cues were presented at the same time as the target of a visual discrimination task but did not carry any information about the task at hand (i.e., orientation discrimination). Importantly, participants either obtained rewards upon correct responses or did not receive any reward feedback in any condition. In the first case, participants’ rewards depended on the identity of auditory or visual stimuli and these cues were *PC* predictors of rewards, whereas in the second case auditory and visual stimuli were *previously associated with rewards* (PR) and did not predict the delivery of reward anymore. We hypothesized the two modes of reward cuing are linked to distinct processes: goal-driven (voluntary) and stimulus-driven (involuntary) attention. In result, when the cues were PC, the voluntary control would dominate and therefore the cues would benefit performance. However, when the cues were associated with rewards in the past and did not lead to reward feedbacks during the test phase, they would only involve the involuntary capture of attention and lead to weaker reward-driven modulations, which may differ between the intra- and cross-modal rewards. Pupil responses can be used as a sensitive readout of changes in the motivational state due to salient events (Chiew and Braver, 2013; Schneider et al., 2018; Pietrock et al., 2019), even when such events are not consciously detected (Bijleveld et al., 2009). Pupil responses have also been recently linked to the level of cognitive effort exerted in a task (van der Wel and van Steenbergen, 2018). We therefore hypothesized that PC reward cues are associated with higher goal-directed cognitive effort in prospect of higher rewards, hence producing a stronger value-driven modulation of pupillary responses compared to cues that were previously associated with rewards.

Our results demonstrate that reward associated cues enhance the accuracy of visual discrimination irrespective of the sensory modality and whether the reward delivery was continued (PC) or halted (PR). Additionally, PC reward cues energized behavior, as indexed by reaction times (RTs) and pupil responses, an effect that was absent in PR cues.

## Materials and methods

### Participants

In total, 43 subjects participated in the experiment to fulfill a target sample size of *N* = 36 based on a previous study (Vakhrushev et al., 2021). They were invited *via* an online recruiting system.^1^ All participants were naïve to the hypothesis of the project, had no history of neurophysiological or psychiatric disorders according to a self-report, had normal or corrected-to-normal vision, and performed the key presses during the task with their dominant hands (five left handed). Eight participants were removed from the final sample, as due to technical problems the experiment had to be terminated before the complete dataset was collected (*N* = 4), the psychometric method used to estimate the orientation discrimination thresholds did not converge on a reliable value (*N* = 2, based on our previous work the QUEST method needed to converge on a stimulus orientation < 2° and performance during the baseline phase needed to be <90%), the participant did not learn the reward associations (*N* = 1) or had a strong bias for one of the colors or sounds prior to learning the reward associations (*N* = 1, estimated as a bias toward high reward colors or sounds > 2.5 SD of the group mean). Thus, the final sample comprised data from 35 participants (18 female; age: 18–45, 27 ± 5 SD years).

Participants were informed that after the experiment they would obtain a reward comprising a fixed hourly rate (∼8 Euros per hours) plus an added bonus that depended on their performance. To calculate the total reward, the fixed hourly rate was added to the money participants obtained during the experiment and a fraction of the total amount (4%) was handed over to the participants in cash.

Before the experiment started and after all procedures were explained, participants gave their oral and written consent. The study was approved by the local ethics committee of the “Universitätsmedizin Göttingen” (UMG), under the proposal number 15/7/15.

### Stimulus presentation and apparatus

**FIGURE 1:**
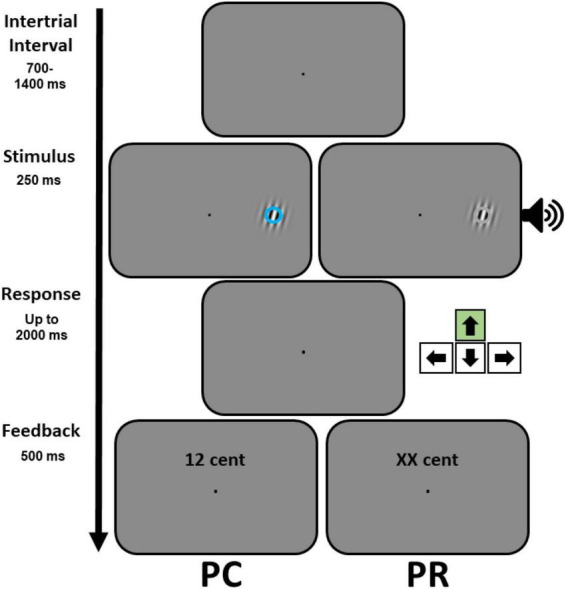
Behavioral paradigm employed during the test phase. An example trial of the visual discrimination task, illustrating the Gabor target and the task-irrelevant visual **(left)** or auditory **(right)** stimuli, is shown. Participants reported the orientation of the Gabor target by pressing either the up or down arrow keys (the correct response for the example trial is illustrated symbolically by the arrow in the green box). Prior to the test phase, participants learned to associate different visual (blue or orange circles) or auditory (high or low pitch tones) stimuli, counter-balanced across participants, with different reward magnitudes during a conditioning phase (see Supplementary Figure 1). The test phase comprised two parts with different reward contingencies (PC and PR). In case of a correct response, during the performance-contingent reward (PC) phase, the monetary reward associated with a specific stimulus was displayed (for instance 12 cent). In a subsequent phase, previously reward-associated (PR) stimuli were not predictive of reward delivery, but to keep the layout of the feedback display similar across the two phases the letters XX were shown for all conditions.

The behavioral paradigms used during the reward associative learning (conditioning) and test phase were identical to a previous study (Vakhrushev et al., 2021). The paradigm employed during the conditioning was a spatial localization task (see Supplementary Figure 1 and the Section “Experimental procedure”) where participants reported the side (left or right) from which visual or auditory stimuli were presented. During the test phase, a visual orientation discrimination task was used in which the tilt direction of a Gabor patch (a Gaussian-windowed sinusoidal grating with SD = 0.33°, a spatial frequency of 3 cycles per degree, subtending 2° diameter, displayed at 9° eccentricity to the left or right side of the fixation point) had to be reported (Figure 1). The tilt orientation of the Gabor patch was set to each participant’s perceptual threshold estimated after the initial training. To determine this threshold, we employed a QUEST algorithm (Watson and Pelli, 1983) to estimate the Gabor tilt orientation for which participants’ performance was at 70%. In each trial, a task-irrelevant semi-transparent ring (alpha 50%, 0.44° in diameter) was superimposed on the Gabor patch. The color of the rings (orange or blue for visual conditions, or gray for auditory and neutral conditions) was adjusted individually for each participant in such a way that they were perceptually isoluminant. Perceptual thresholds for the visual discrimination task were determined when Gabors were superimposed with a gray circle. For auditory cues, two pure tones with different frequencies (350 or 1,050 Hz) were presented at 70 dB simultaneously with the Gabor patch and at the same side.

The timing of events was identical across the experiment (see Figure 1 and Supplementary Figure 1). As soon as participants fixated (within 1° of the fixation point) a trial started. After an additional fixation period of 700–1400 ms, a target stimulus appeared (either a colored circle or a tone during conditioning or a Gabor patch together with a colored circle or a tone during the test phase). The target stimulus disappeared after 250 ms and participants had to indicate its side (conditioning) or the orientation of the Gabor patch (during the test phase) within 2,000 ms from the onset of the target. Finally, a feedback display was presented for 500 ms. The feedback display contained the reward magnitude that participants received (in numbers) during conditioning and PC phase (see the Section “Experimental procedure”). To keep the visual layout of the feedback display similar across PC and PR phases, in the latter phase “xx cent” was shown for all conditions.

Throughout the experiment, visual stimuli were displayed on a calibrated ViewPixx monitor (refresh rate = 120 Hz, resolution 1,080 × 1,920 pixels, and placed at a viewing distance of 60 cm). The auditory tones were delivered through an over-ear headphone (HAD 280 audiometry headphones, Sennheiser).

### Experimental procedure

The experiment consisted of a practice session (32 trials) for the orientation discrimination task and three phases. In the first phase, referred to as the *baseline phase* (160 trials), participants were required to report the tilt direction of a Gabor patch relative to the horizontal meridian by pressing a keyboard button (either the down or up arrow keyboard button for clockwise and counter-clockwise directions, respectively; see Figure 1). They were additionally instructed to ignore the simultaneously presented visual or auditory cues that accompanied the Gabor. Afterward, participants completed a *conditioning* task to learn the reward associations of auditory and visual cues (see Supplementary Figure 1). In this task, participants decided whether a colored circle or an auditory tone was perceived to be on the left or right side by pressing the corresponding arrow key buttons. Upon correct response, participants saw the magnitude of the reward that was paired with a certain cue and thereby learned whether a visual or auditory stimulus was associated with high (mean = 25 Cents) or low (mean = 2 Cents, drawn from a Poisson distribution) monetary reward. In the third phase, referred as the *test phase*, participants performed the same orientation discrimination task as in the baseline phase, but in the presence of task-irrelevant visual or auditory cues that had been associated with different amounts of reward during conditioning. As the main task was a visual discrimination task, task-irrelevant visual and auditory stimuli will be referred to as intra- and cross-modal, respectively. Additionally, the test phase was split into two parts: in the first part (320 trials, the phase with *performance-contingent reward cues*, *PC*), upon correct response, similar reward feedbacks as in the conditioning phase were presented, i.e., reward depended on the identity of cues and was either high or low. In the second part (320 trials, referred to as the phase with *previously associated reward cues*, *PR*), the delivery of rewards was halted. Here, participants were instructed similarly to the PC phase with the exception that they were informed about a different feedback display shown after each trial. Specifically, they were told that in the PR phase the differential reward deliveries would be halted and instead after each trial they would see a feedback in the form of “xx cent” indicating a constant amount of reward that would be added to their total earning in case they responded correctly.

In order to determine whether participants learned the reward-cue association, they were asked to indicate which cue from each modality presented to them sequentially had been associated with more money. This question was completed in multiple parts following the conditioning, PC, and PR phases. Additionally, we also repeated the question in the questionnaire after the experiment was completed. If a participant did not provide any correct response across all experimental phases (*conditioning*, PC and PR), then the participant was removed from further analysis (*N* = 1).

### Pupillometry

An EyeLink 1000 Plus system with a desktop mount (SR Research) was used to track the right eye. The EyeLink camera was controlled by the corresponding toolbox in MATLAB (Cornelissen et al., 2002). Before each block, the eye tracking system was calibrated using a nine-point standard EyeLink calibration procedure.

Pupil responses were acquired at a sampling frequency of 1,000 Hz. The pupil data of each trial was extracted from 100 ms prior to the target onset until the end of the trial (i.e., the end of the feedback display). Trials in which more than 50% of data was lost were removed from further analysis. For the missing data due to blinks, a linear interpolation was applied, where the missing data was interpolated based on the samples within a window of 10 ms before and after the blink. The data was then low-pass filtered (fourth order Butterworth with a cut-off frequency of 2 Hz), normalized to z-score (across all samples recorded for each participant) and subsequently corrected for baseline (i.e., 100 ms). For the statistical analysis, the average stimulus-evoked response in a window from the target onset until the end of each trial (the end of the feedback display as shown in Figure 1) was examined. Note that a trial’s timing depended on how fast the participant responded. Therefore, to examine the relation between the pupil size and the behavioral measures, pupil responses were estimated from the data of the first 500 ms interval after the target onset. This was done to ensure that for all participants and all experimental conditions the same number of pupil samples were considered.

### Data analysis

The data obtained from all parts of the experiment was analyzed using custom-written scripts in MATLAB (version R2015a). We analyzed accuracies, reaction times (RT: median reaction time across correct and incorrect trials), inverse efficiency scores (IE) (median RT of correct trials divided by the accuracy) d-prime (d′) and pupil size. We removed trials in which any of the following conditions were met: lack of stable fixation during the presentation of the target (i.e., the distance of eye gaze from the fixation point exceeded 0.9°), no response, RTs exceeding the 2.5 SD of each phase, or loss of more than 50% of pupil data. This resulted in 2.98% (±1.20 SD), 2.62% (±2.25 SD), 3.01% (±1.04 SD), and 3.64% (±2.97 SD) trials removed from baseline, conditioning, PC and PR phases, respectively. For each response variable, we calculated the average across all trials of each condition per subject during the baseline and test phases separately. D-prime was measured based on the probability of hits and false-alarms, as d′ = Z(PHit)–Z(PFA), where one of the tilt directions was arbitrarily treated as “target-present” as in formal Signal Detection Theory analysis of discrimination tasks (Macmillan and Creelman, 1991). Extreme values of PHit or PFA were slightly up- or down-adjusted (i.e., a probability equal to 0 or 1 was adjusted by adding or subtracting $\frac{1}{2\times N}$, where *N* is the number of trials, respectively). Afterward, the difference in response variables (accuracies, reaction times, d′ and pupil size) between baseline and test phase was entered to a 2 × 2 × 2 repeated measures ANOVA, with the reward contingency (performance-contingent: PC and previously associated: PR), reward magnitude (high and low), and sensory modality (visual or auditory, i.e., intra- and cross-modal, respectively) as within-subjects factors. Significant effects in RM ANOVA were followed up by *post-hoc* tests (*multcompare* in MATLAB with *Bonferroni* correction). To test whether the value-driven modulation of pupil size is predictive of the modulation of the behavioral measures a robust regression method (*robustfit* with default settings in MATLAB) was employed.

## Results

The main objective of this study was to examine whether visual discrimination is influenced by co-occurring visual and auditory stimuli which did not carry any information about the dimension over which the discrimination was performed (i.e., the orientation of a Gabor stimulus, see Figure 1) but were either predictive of the reward delivery upon correct performance (i.e., performance-contingent: PC phase) or were previously associated with the reward delivery (i.e., previously rewarded: PR phase). Participants first learned the reward associations of visual and auditory stimuli during a conditioning phase by performing a localization task (see the Supplementary Text and Supplementary Figure 1). We found a weak effect of reward on the behavioral performance and pupil responses (see the Supplementary Text and Supplementary Figure 2) during the conditioning phase. Nevertheless, the conditioning task was successful in establishing the associations between stimuli and rewards, as according to the debriefings performed after this phase, all participants had learned the reward associations of tones and colors correctly. Therefore, we next examined the behavioral and pupillometric responses during the visual discrimination task, testing whether the learned reward associations affected the visual perception during the PC and PR phases compared to the baseline (i.e., done prior to the conditioning).

### Effect of performance-contingent and previously associated reward cues on the accuracy of visual discrimination

**FIGURE 2:**
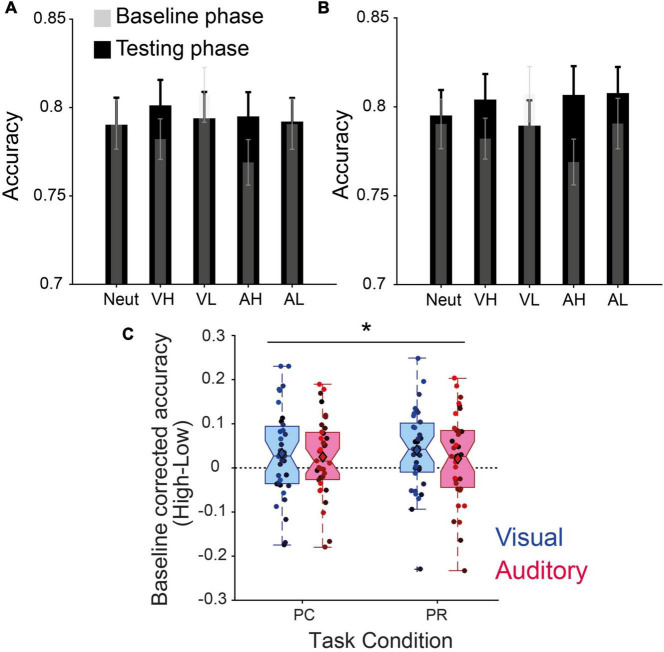
Value-driven modulation of discrimination accuracy. **(A)** Accuracies of the baseline and performance-contingent reward (PC) phase. **(B)** Same as panel **(A)** for the previously associated rewards (PR) phase. The transparent gray shades represent the baseline performance before learning the reward associations, overlaid on the test phase performance in black for each condition (neut, neutral; VH, visual high-; VL, visual low-; AH, auditory high-; and AL, auditory low-reward). **(C)** Baseline -corrected reward effect (high–low) for intra-modal (visual) and cross-modal (auditory) reward cues during the two phases. Error bars in panels **(A,B)** represent s.e.m., circles with different color shades in panel **(C)** correspond to the data of individual participants, and * stands for the main effect of reward at *p* < 0.05.

Overall, during the initial baseline phase where the cues were not associated with any reward magnitude, participants performed on average across all conditions with 78.78% accuracy (±0.94 s.e.m) (Figures 2A, B), while in the PC phase, mean accuracy increased to 79.44% (±1.23 s.e.m) and in the last phase with PR cues increased to 80.06% (±1.32 s.e.m). This indicated that with time, participants became more proficient in the task. However, the improvement of accuracy across time (Baseline, PC and PR) did not reach statistical significance [*F*(2,34) = 1.04, *p* = 0.35, η_p_^2^ = 0.03].

In the test phase, a repeated measures 2 × 2 × 2 ANOVA conducted on the baseline corrected accuracy rates showed a significant main effect of reward magnitude across PC and PR phases (Figure 2C): *F*(1,34) = 7.37, *p* = 0.01, η_p_^2^ = 0.18. All other main and interaction effects were non-significant (all *p*s > 0.1). *Post-hoc* tests revealed a significant increase in accuracies by high- compared to low-reward visual cues in PR (*p* = 0.016, Cohen’s d = 0.430), a trend in PC (*p* = 0.068, Cohen’s d = 0.319) and non-significant effects in auditory conditions (PC: *p* = 0.108, Cohen’s d = 0.279; and PR: *p* = 0.235, Cohen’s d = 0.204). We obtained similar results when d-prime (d′) scores instead of accuracies were used [*F*(1,34) = 6.75, *p* = 0.01, η_p_^2^ = 0.17], indicating that the improvement in participants’ performance was not driven by an enhanced false-alarm rate.

The main effect of reward is in line with our hypothesis predicting that high-reward cues improve the perceptual discriminability. Contrary to our predictions, we did not find a significant interaction effect with reward contingency or sensory modality, although the effect sizes were larger for intra-modal (visual) cues.

### Effect of performance-contingent and previously associated reward cues on the speed of visual discrimination

**FIGURE 3:**
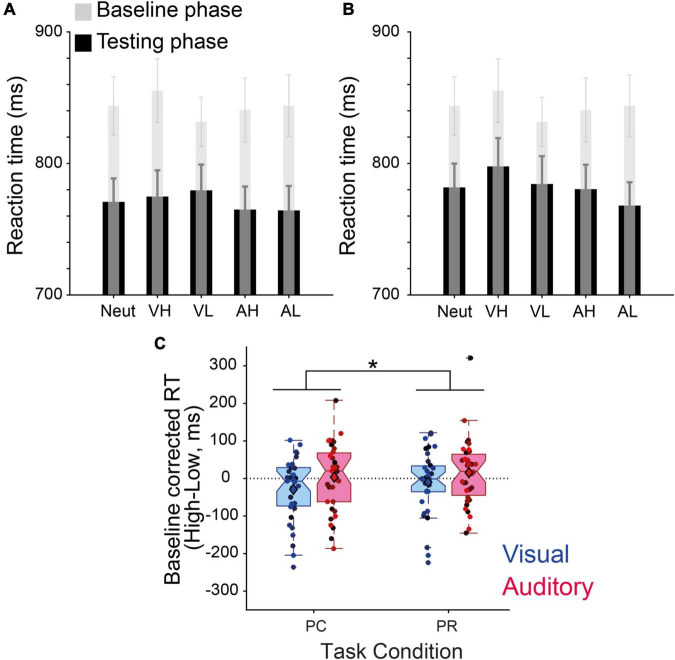
Value-driven modulation of discrimination speed. **(A)** Reaction times (RTs) (ms) of the baseline and performance-contingent reward (PC) phase. **(B)** Same as panel **(A)** for the previously associated rewards (PR) phase. The transparent gray shades represent the baseline RTs before learning the reward associations, overlaid on the test phase performance in black for each condition (neut, neutral; VH, visual high-; VL, visual low-; AH, auditory high-; and AL, auditory low-reward). **(C)** Baseline–corrected reward effect (high–low) for intra-modal (visual) and cross-modal (auditory) reward cues during the two phases. Error bars in panels **(A,B)** represent s.e.m., circles with different color shades in panel **(C)** correspond to the data of individual participants, and * stands for the interaction effect between reward and task phase at *p* < 0.05.

The analysis of RTs across all conditions demonstrated that participants became overall faster as they proceeded through the experiment (Figures 3A, B), an effect that reached statistical significance when tested with an ANOVA with phase (Baseline, PC and PR) as the independent factor [*F*(2,34) = 21.39, *p* < 10^–7^, η_p_^2^ = 0.39]. Participants’ RTs in both PC (*M* = 770.83 ms, s.e.m = 18.24 ms) and PR phases (*M* = 782.41 ms, s.e.m = 18.93 ms) were significantly faster than the baseline phase (*M* = 843.01 ms, s.e.m = 21.33 ms, both *p*s < 10^–4^).

A repeated measures 2 × 2 × 2 ANOVA on the baseline corrected RTs revealed a significant interaction between reward magnitude and task contingency [*F*(1,34) = 4.61, *p* = 0.039, η_p_^2^ = 0.12, Figure 3C]. This effect demonstrates that when cues associated with higher value were predictive of the reward delivery, participants reacted faster than when reward delivery was halted. Specifically, *post-hoc* tests revealed that this effect was more pronounced for PC, high-reward visual cues (*p* = 0.048, Cohen’s d = 0.33) than other conditions (visual/PR: *p* = 0.47, Cohen’s d = 0.123; auditory/PR: *p* = 0.30, Cohen’s d = 0.178; auditory/PC*: p* = 0.80, Cohen’s d = 0.043). Although mostly driven by the visual cues, this finding is in line with our hypothesis predicting that PC rewards have a stronger influence on the speed of perceptual decisions.

### Effect of performance-contingent and previously associated reward cues on pupil responses

**FIGURE 4:**
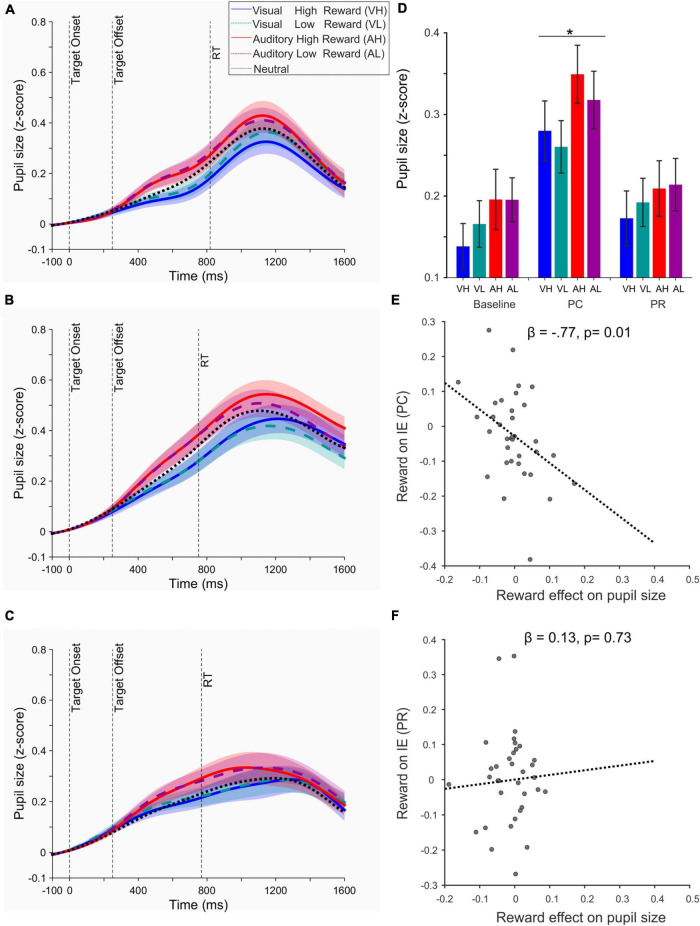
**(A)** Time course of pupil response for each condition during the baseline phase. **(B)** Same as panel **(A)** during the performance-contingent phase (PC). **(C)** Same as panel **(A)** during the previously associated rewards (PR). In panels **(A–C)** the vertical dashed line denoted as RT shows the mean reaction time across all conditions and across all participants. **(D)** Bar plots represent the mean task-evoked pupil size measured from the target onset until the trial end (i.e., the end of feedback phase, see Figure 1) for each condition (VH, visual high-; VL, visual low-; AH, auditory high-; and AL, auditory low-reward). *The effect of reward value was only significant in the PC phase at *p* < 0.05. **(E)** Relation of the value-driven modulation of pupil size (in the first 500 ms after the target onset) and inverse efficiency scores (IE) during the PC phase. **(F)** Same as panel **(E)** during the PR phase. In panels **(E,F)** regression lines are estimated based on a robust regression analysis.

We next examined the pupil responses using a 2 × 2 × 2 repeated measure ANOVA with three factors: reward magnitude (high and low), sensory modality (auditory and visual), and reward contingency (performance-contingent: PC and previously associated: PR). Pupil responses were the baseline corrected average pupil size (z-score) extracted from the target onset until the trial end (Figure 4). Across all visual and auditory conditions, task-evoked pupil responses were significantly higher in PC compared to PR phase [*F*(1,34) = 61.32, *p* < 10^–8^, η_p_^2^ = 0.643]. Additionally, a significant interaction effect was observed between the reward magnitude and contingency [*F*(1,34) = 7.17, *p* = 0.011, η_p_^2^ = 0.174], as higher rewards increased the pupil size compared to lower rewards only in PC (*p* = 0.04, Cohen’s d = 0.354) but not in PR phase (*p* = 0.94, Cohen’s d = 0.014). A weaker interaction effect [*F*(1,34) = 4.80, *p* = 0.035, η_p_^2^ = 0.124] was also observed between the sensory modality and reward contingency, corresponding to larger pupil responses evoked by cross-modal (auditory) compared to intra-modal (visual) stimuli in PC phase and an opposite effect in PR phase. The effect of sensory modality in each phase did not reach significance (PC: auditory-visual = 0.02 ± 0.02 s.e.m, *p* = 0.31; PR: auditory-visual = −0.01 ± 0.02 *p* = 0.34, *p* = 0.34).

The lack of reward-driven effects in the PR phase could be due to a time-dependent habituation of pupil responses to reward rather than the termination of reward delivery, since the PR phase consistently occurred after the PC phase. However, we ruled out this possibility by examining the pupil responses of the first and second half of each phase (see the Supplementary Text and Supplementary Figure 3).

We next examined whether the value-driven modulation of pupil responses observed in the PC phase exhibited any correlation with the modulation of our behavioral measures. Since we observed both a modulation of accuracy (Figure 2) and RTs (Figure 3), we combined these measures into one single parameter, i.e., IE defined as the ratio of RTs of correct trials to accuracy (Vandierendonck, 2021). This parameter provides a measure of how well participants adjust their speed-accuracy trade-off. We found a strong linear relation (β = −0.77, *t*_33_ = −2.59, *p* = 0.01, Figure 4E) between the net effect of reward on pupil size (i.e., pupil size in high reward condition of both modalities minus pupil size in low reward of both modalities) and on IE scores. This effect indicates that a stronger value-driven pupil dilation was predictive of a stronger value-driven acceleration of visual discrimination across participants. This correlation was non-existent in the PR phase (β = 0.13, *t*_33_ = 0.35, *p* = 0.73, Figure 4F).

## Discussion

This study aimed to compare PC and previously associated (PR) reward cues from visual or auditory modality in terms of their modulatory effects on visual perception and task-evoked pupil responses. Our results showed that reward associated cues exert a persistent effect, in that once the reward associations are learned, reward cues improved the accuracy of perceptual judgments even when rewards were not delivered anymore (i.e., during the PR phase). PC cues were overall associated with larger task-evoked pupil responses indicating that they invoke more engagement with the task and higher goal-driven control. Furthermore, in contrast to PR, PC cues especially in visual modality, also sped up perceptual choices when a higher reward was expected and this effect was correlated with the value-driven modulation of pupil responses. These results suggest that despite the persistent effects of reward even when reward delivery is halted, some aspects of value-driven effects are specific to PC cues.

Previous research has provided evidence for a value-driven modulation of perception when the task-relevant features of stimuli are associated with high reward (Chelazzi et al., 2013; Pessoa, 2015; Failing and Theeuwes, 2018), an effect that also persists when the reward delivery is halted (De Tommaso et al., 2017). Conversely, it has been shown that the association of task-irrelevant stimuli with rewards inflicts a cost on performance, likely due to capturing attention away from the target and exhausting the cognitive control mechanisms (Sali et al., 2013; Anderson et al., 2014; Rusz et al., 2020; Watson et al., 2020). Although the majority of past research has focused on visual modality, recent studies have also examined the cross-modal effects of rewards (Leo and Noppeney, 2014; Pooresmaeili et al., 2014). Interestingly, the latter studies showed that cross-modal (auditory) stimuli that have been previously associated with higher rewards facilitated visual perception compared to low reward stimuli, despite being irrelevant to the task at hand. These findings suggest that the value-driven increase in the salience of task-irrelevant stimuli is not necessarily associated with costs on performance. What determines whether rewards boost or impair perception in light of findings of the current study and the past research?

To understand the divergent effects observed across studies and thereby provide a unifying explanation for reward effects on perception, it is important to point to differences in the design and experimental procedures that were employed. There is a critical difference between the current study and previous studies showing that task-irrelevant reward cues captured attention away from the target and were thus associated with a cost on performance (Sali et al., 2013; Anderson et al., 2014; Rusz et al., 2020; Watson et al., 2020). In those previous studies, the majority of which employed a visual search paradigm, the target and the reward associated task-irrelevant stimuli were spatially separated. This separation might be the factor explaining the capture of attention to a different location than the target by reward cues, thereby competing with the task goal. In our study, however, both target and task-irrelevant reward cues were presented at the same spatial location, hence the capture of attention by task-irrelevant high reward cues may have spilled over to the target, increasing its representation and therefore optimizing behavior compared to low reward cues. This is in line with the findings of MacLean and Giesbrecht (2015) showing that when task-irrelevant cues were in the same location as the probed target, cues associated with higher reward magnitude improved visual search performance compared to low reward magnitude. Another related possibility is that higher reward may in fact promote perceptual grouping between the reward- associated cues and the target, as reward has been shown to interact with object-based attention (Shomstein and Johnson, 2013; Stanisor et al., 2013; Zhao et al., 2020). Therefore, in our paradigm high-reward task-irrelevant cues may have enhanced the processing of the target through a combination of space-based and object-based selection mechanisms, especially since during the PC phase these cues were predictive of the reward delivery.

The results of the current study show that PR stimuli can have long-lasting facilitatory effects on perception. However, we note that PR phase in our experiment was only tested after the PC phase, and therefore participants had a long exposure to the reward cues in a setting when they were predictive of the reward delivery when orientation discrimination task was performed correctly (i.e., the PC phase). In contrast, in our previous work (Vakhrushev et al., 2021), we tested the PR phase only after a conditioning phase which employed a different task (i.e., cue localization) than the test phase (i.e., orientation discrimination), and found that reward effects were most prominent for cross-modal cues. Together, the current results and results of our previous study indicate that the effects of reward critically depend on the training mode (Jahfari and Theeuwes, 2017; Failing and Theeuwes, 2018) and the relation between the rewarded stimuli and the task-relevant features.

Although accuracies were enhanced by high reward cues in both PC and PR phase, speed of visual discrimination was only modulated by rewards in the PC phase, especially for intra-modal cues. It is important to note that our task instructions encouraged accuracy over speed, as participants received a reward only for correct responses and independent of RT. Therefore, speeding up choices in PC occurred without an explicit instruction for speedy responses or an impact of doing so on reward magnitudes. However, by increasing the speed of choices during the PC phase for high reward cues, participants could increase their total reward rate, i.e., the amount of reward obtained in a given time for a self-paced task, a factor that has been shown to play an important role in perceptual decision making (Gold and Shadlen, 2002). When reward delivery is halted increasing the reward rate is not at stake anymore and hence in PR we did not find a speed enhancement. The motivation to increase speed in high reward PC trials, however, did not lead to a decrement in accuracy due to speed-accuracy-trade-off, suggesting that the goal-driven control mechanisms invoked by PC cues may increase the overall efficiency of perceptual choices.

Examination of pupil responses provided further evidence that PC reward cues invoke a stronger engagement of goal-driven mechanisms, as demonstrated by two key findings. Firstly, we found a stronger task-evoked pupil dilation in PC across all conditions, suggesting that in this phase participants exerted overall higher cognitive effort compared to the PR phase. Recruiting higher cognitive effort is known to increase the activity of noradrenergic neurons in Locus Coeruleus (LC) and thereby induce pupil dilation (van der Wel and van Steenbergen, 2018). Accordingly, previous studies have shown that large pupils predict the higher cognitive control required before goal-directed eye movements (Mathôt et al., 2015), reflect the higher effort required for task switching (da Silva Castanheira et al., 2021), and are indicative of the degree to which endogenous orientating of spatial attention is invoked by a task (Lasaponara et al., 2019). Importantly, the degree to which humans engage in a cognitively effortful task depends on the inherent relation between costs and benefits that ensue from performing a task (Shenhav et al., 2021) and whether the cost-benefit relations remain predictable over time (Manohar et al., 2017). In our experiment, the continuous and consistent delivery of reward upon correct performance in PC may have allowed a more direct estimation of how much rewards could compensate for the cost of extra cognitive effort, hence encouraging participants to maintain a sustained heightened level of goal-directed attention across all conditions. Secondly, in addition to the overall heightened dilation of pupils in PC phase, we found that only in this phase value-driven modulation of pupil size was significant, and this effect was predictive of the behavioral speed modulation. Modulation of pupil responses by reward value is in line with a number of previous findings (Chiew and Braver, 2013, 2014; Massar et al., 2016; Koelewijn et al., 2018; Pietrock et al., 2019; Walsh et al., 2019) and indicates that when the delivery of reward is contingent on task performance, higher reward incentives could efficiently mobilize the processing resources, and settle an efficient relationship between the speed and accuracy of choices, effects that are also reflected in the task-evoked pupil dilatation and have been reported across motor (Naber and Murphy, 2020), perceptual (Walsh et al., 2019), and cognitive (Kozunova et al., 2022) tasks. On the other hand, the lack of value-driven modulation of pupil responses for PR cues is in line with effects reported in previous studies, where reward-driven modulations of pupil size were only found during the learning of reward associations (Anderson and Yantis, 2012) but were absent during the test phase when reward-associations were implicit (Hammerschmidt et al., 2018). Taken together, these findings suggest that pupillary responses are not modulated by the mere exposure to the associative value of stimuli, but rather depend on the context in which rewards are delivered (Preuschoff et al., 2011; Cash-Padgett et al., 2018).

In the current study, the PR phase consistently occurred after the PC phase. Although our results in the PR phase could be directly compared to the previous studies that used a similar design (Vakhrushev et al., 2021), future studies would benefit from counterbalancing the task order across participants to confirm whether the results in each phase and the differences observed between PC and PR phases could be replicated. In fact, comparing our results to those reported previously (Vakhrushev et al., 2021), suggests that the reward-driven effects in the PR phase, especially for intra-modal cues, could be boosted when preceded by a phase when the delivery of rewards is PC, although this conclusion awaits future replications. In doing so, future studies may also benefit from using a larger sample size, as across experiments the effect sizes that we observed were relatively small. However, we also notice that small effect sizes could be due to the nature of the task we employed, as unlike previous studies, we used reward cues that did not carry information about the target of the visual discrimination task, a scenario when rewards and attentional requirements of the task align and larger reward driven effects are expected. Furthermore, studies on pupillometric correlates of value-driven effects can make use of paradigms in which the timing of events in each trial is tailored to the sluggish nature of pupil responses. Specifically, in our study the trial duration was relatively short (1,450–2,150 ms), which might have been insufficient to isolate the sluggish pupil modulations evoked by some of the conditions. This can be achieved by introducing a delay between the target offset and the appearance of the feedback display (see Figure 1) and by prolonging the intertrial intervals (ITI). Another important direction for future studies would be to further investigate which neural mechanisms give rise to the behavioral and pupillary effects that were observed here, through using neuroimaging or electrophysiological methods. This direction is important as it will allow to test whether the stronger involvement of goal-driven control during PC phase occurs through the same mechanisms that underlie attentional and reward-driven selection, namely, an enhanced engagement of fronto-parietal attentional regions (Corbetta and Shulman, 2002; Padmala and Pessoa, 2011) or changing the temporal profile of attentional control (Krebs et al., 2013). Moreover, future neuroimaging studies should investigate how the sensory modality of rewards interacts with the value-driven modulations of perception, as intra-modal and cross-modal reward effects may rely on distinct neural mechanisms (Vakhrushev et al., 2021).

In summary, our findings demonstrate a persistent effect of intra- and cross-modal rewards on visual perception. The stronger goal-driven control invoked by PC rewards and reflected in pupil responses, can additionally enhance the overall efficiency of perceptual choices by increasing the speed without sacrificing the accuracy.

## Data availability statement

The raw data supporting the conclusions of this article will be made available by the authors, without undue reservation.

## Ethics statement

The studies involving human participants were reviewed and approved by Local Ethics Committee of the “Universitätsmedizin Göttingen” (UMG), under the proposal number 15/7/15. The patients/participants provided their written informed consent to participate in this study.

## Author contributions

JA and AP conceptualized the project, interpreted the results, and wrote the first draft of the manuscript. JA conducted the experiments. AP acquired funding. All authors designed the task, analyzed the data, and revised the manuscript.

## Footnotes

1
http://www.probanden.eni-g.de/orsee/public/
