# Supplementary material for: Value-driven modulation of visual perception by visual and auditory reward cues: The role of performance-contingent delivery of reward
Source: Front Hum Neurosci. 2022 Dec 23;16:1062168. doi: 10.3389/fnhum.2022.1062168 (PMC9816136; doi:10.3389/fnhum.2022.1062168)
Supplement: Supplementary file 1 [file Data_Sheet_1.pdf]

Supplementary Information to: “Value-driven modulation of visual perception by visual and auditory reward cues: the role of performance-contingent delivery of reward”

Jessica Emily Antono, Roman Vakhrushev, Arezoo Pooresmaeili

Supplementary Figures

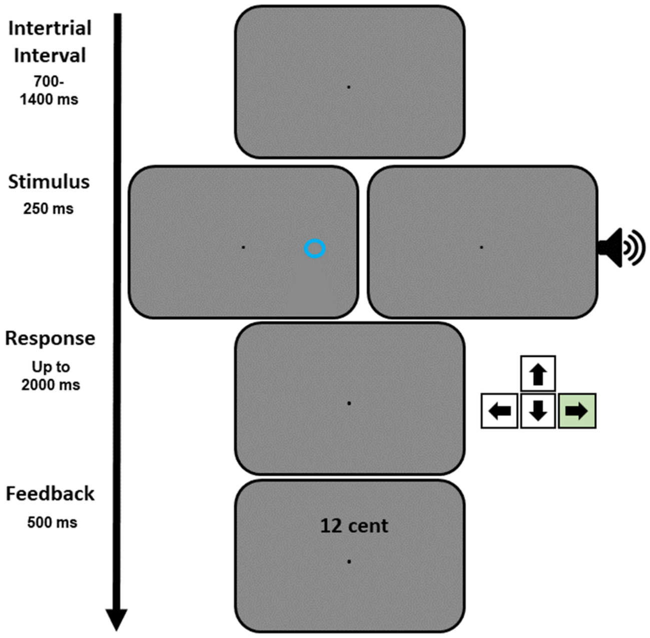

Figure S1. Behavioral paradigm employed during the conditioning phase. Participants were asked to report whether a visual (left) or an auditory (right) stimulus was presented to the left or right side and indicate their responses by pressing the corresponding left or right arrow keys on the keyboard. Here, two example trials are shown in which the stimulus is presented to the right side and hence the correct response was to press the right arrow key (highlighted arrow box in green). In case of a correct response, the monetary reward associated with a specific stimulus was displayed (for instance 12 cent), and thereby participants learned the reward associations of different colors and auditory tones.

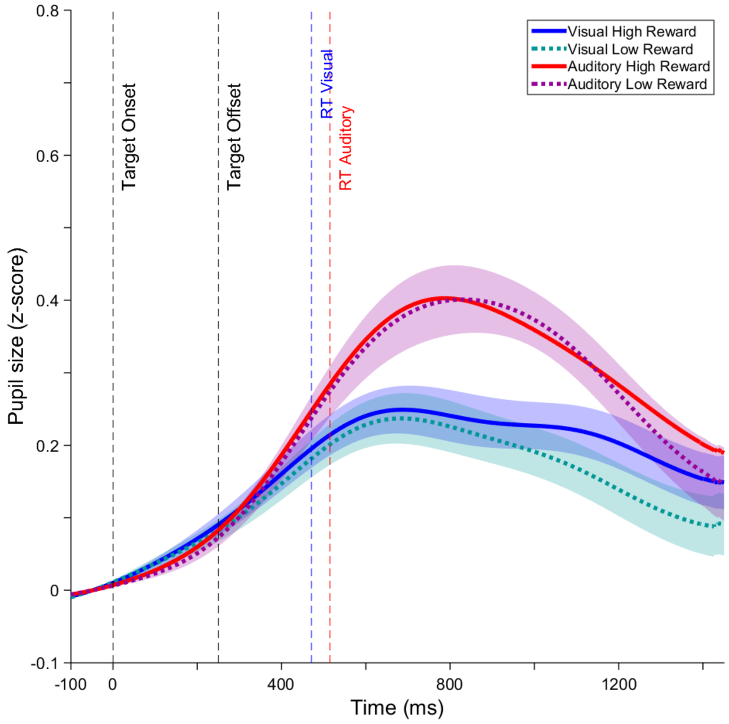

Figure S2. Pupil responses during the conditioning phase. The vertical dashed light in blue shows the mean reaction time across all visual cues across all participants and the red dashed line shows the mean reaction time across all auditory cues across all participants. For more details see the section "Behavioral and pupillometry results during the conditioning" in the Supplementary Text.

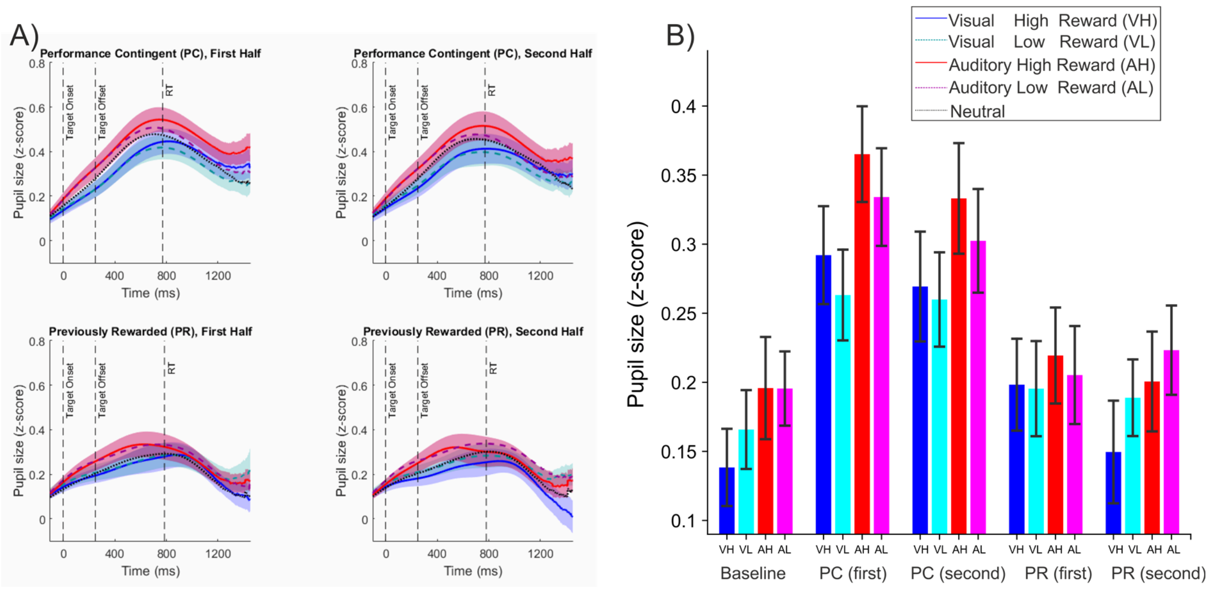

Figure S3. Pupil responses in the first and second half of the performance-contingent (PC) and previously rewarded (PR) phases. A) Time course of pupil responses in the first and second half of PC phase (upper row) and in the first and second half of PR phase (lower row). The vertical dashed line denoted as RT shows the mean RT across all conditions and across all participants. B) Bar plots represent the mean task-evoked pupil size measured from the target onset until the trial end for each condition (VH: visual high-, VL: visual low-, AH: auditory high-, and AL: auditory low-reward) for different phases. The data in PC and PR phases is separately shown for the first and second half of all trials (as in A). For more details see the section "Examining the changes in reward-driven effects on pupil responses with time" in Supplementary Text and Figure 4 in the main text.

Supplementary Text

Behavioral and pupillometry results during the conditioning

During the conditioning, participants performed a localization task and thereby learned the reward associations of the tones and colors, as each correct response led to a monetary reward that depended on the identity of stimuli (see the Material and Methods and **Figure S1**). Participants' overall performance in this task was nearly perfect for both modalities (99.75% ± 0.11 s.e.m and 99.53% ± 0.21 s.e.m for visual and auditory stimuli, respectively), with no significant difference between the modalities ($F(1,34) < 1$), as intended. Additionally, a small but significant main effect of reward value on increasing the localization accuracies (by 0.02% ± 0.17 s.e.m and by 0.61% ± 0.25 s.e.m for high compared to low reward cues in visual and auditory modality, respectively, $F(1,34) = 4.43$, $p = 0.04$, $\eta_p^2 = 0.12$) and a trend for an interaction with modality ($F(1,34) = 3.74$, $p = 0.06$, $\eta_p^2 = 0.10$) were found.

Analysis of reaction times (RTs) only revealed a significant main effect of modality ($F(1,34) = 40.76$, $p = 0.28$, $\eta_p^2 = 0.55$) corresponding to the faster RTs for the localization of visual stimuli compared to the auditory tones (472.22 ms ± 16.80 s.e.m and 517.11 ms ± 16.95 s.e.m,

respectively), an effect that is in line with the superior performance of vision for spatial localization (Welch and Warren, 1980). Although in both modalities response times were decreased for high compared to low reward stimuli (-2.34 ms ± 6.48 s.e.m, and -7.60 ms ± 5.14 s.e.m, for the decrease of RTs in visual and auditory high reward cues, respectively), this effect did not reach statistical significance ($F(1,34) = 1.203$, $p = 0.28$, $\eta_p^2 = 0.03$) and no interaction was found between the reward effect and the stimulus modality ($F(1,34) < 1$).

Analysis of pupil size during conditioning (Supplementary **Figure S2**), revealed a main effect of modality ($F(1,34) = 18.23$, $p < 0.001$, $\eta_p^2 = 0.35$) corresponding to a larger pupil size in response to auditory compared to visual stimuli and a smaller main effect of reward ($F(1,34) = 4.43$, $p = 0.046$, $\eta_p^2 = 0.11$). The effect of reward did not reach significance in individual sensory modalities (ps>0.1).

Overall, we found a small effect of rewards on increasing the localization accuracies and increasing the task-evoked pupil responses but no effect on the performance speed (RTs). The lack of a behavioural effect of rewards on RTs during the conditioning is likely due to the fact that the localization of visual and auditory stimuli was done as a nearly perfect level.

Examining the time-dependence of reward-driven effects on the pupil responses

We observed a significant reward-driven enhancement of pupil responses only during the performance-contingent (PC) phase. The lack of reward effects during the previously rewarded (PR) phase could potentially be due to a time-dependent habituation of pupil responses to reward, as PR phase consistently happened after the PC phase, and not due to the termination of reward delivery. To rule out this possibility, we divided the trials in each phase to two halves and examined whether the reward-driven effects were different across time (**Figure S3**). An ANOVA on pupil responses during the performance contingent phase revealed only a main effect of reward value ($F(1,34) = 4.20$, $p = 0.048$, $\eta_p^2 = 0.11$), but no interaction with time ($F(1,34) = 0.33$, $p = 0.57$, $\eta_p^2 = 0.01$). In a second analysis, we entered the pupil data of both halves of the two phases to the analysis. This analysis reproduced our results reported in the main text when all trials were included: we again observed a significant interaction between the reward value and phase ($F(1,34) = 6.28$, $p = 0.017$, $\eta_p^2 = 0.01$) corresponding to a stronger reward-driven modulation in the performance-contingent phase and importantly no interaction ($F(1,34) = 1.51$, $p = 0.227$, $\eta_p^2 = 0.043$) of this effect with time (first or second half of the data).

These results thus rule out the possibility that the lack of reward effects in the previously rewarded phase is solely driven by a time-dependent habituation to reward delivery.

Citations

Welch, R. B., and Warren, D. H. (1980). Immediate perceptual response to intersensory discrepancy. *Psychol. Bull.* 88, 638–667. doi: 10.1037/0033-2909.88.3.638.
